# "The Hospital" Medical Book Supplement.—No. XVIII

**Published:** 1909-05-22

**Authors:** 


### "THE HOSPITAL" MEDICAL BOOK SUPPLEMENT.—No. XVIII.

CONTENTS.

**Surgery—**
A Manual of Operative Surgery... ... ... ... ... ... ... 215
Gunshot Wounds ... ... ... ... ... ... ... ... ... 215
**Gynæcology—**
Text-book of Gynæcological Diagnosis ... ... ... ... ... 216
Essays on the Position of Abdominal Hysterectomy in London 216
**Tropical Medicine—**
Review of Recent Advances in Tropical Medicine, Hygiene, and Veterinary Science ... ... ... ... ... ... ... 216
**Pædiatrics—**
Infant Feeding ... ... ... ... ... ... ... ... ... 217
**Eugenics and Heredity—**
Heredity and Disease ... ... ... ... ... ... ... ... 217
The Eugenics Review ... ... ... ... ... ... ... ... 217
**Miscellaneous Items—**
Medical Reports of the Central London Throat and Ear Hospital 218
International Clinics ... ... ... ... ... ... ... ... 218
A History of the Reading Pathological Society ... ... ... 218
The Sportsman's Guide to North-Eastern Rhodesia ... ... 218
The "Nauheim" Treatment of Diseases of the Heart and Circulation... ... ... ... ... ... ... ... ... 218

### SURGERY.

A Manual of Operative Surgery. By Sir Frederick Treves, Bart., and Jonathan Hutchinson. Vol. I. (London: Cassell and Company. Third Edition. 1909. Price 18s. net.)

Although ostensibly only a new edition, this is to all intents and purposes a new book. Mr. Hutchinson has impressed his personality upon the style and character of the letterpress, has added illustrations—many of them in colour—which greatly enhance the value of the verbal descriptions, and has achieved, we think, his professed aim to preserve "everything of permanent value in previous editions" and, at the same time, to increase its practical utility. He certainly has adhered to Treves' original intention of making no attempt at encyclopædic completeness, but no pains have been spared to ransack the literature—at any rate English literature—so as to ensure the inclusion of most advances in technique of proved importance. There is a healthy dogmatism about a good many statements in the text that will induce the holders of other views to examine the foundation of their own surgical beliefs, without offending their susceptibilities. Despite the prominence most properly given to descriptions of modern theatre conditions, and of ceremonial observances in the temple of Asepsis, there is throughout a noticeable conservatism. It would be out of the question to take the various sections *seriatim*, and although any surgeon could find something with which to disagree in almost any one of them, it would be over some matter of minor importance or detail. Take for example the account of pylorectomy; probably few surgeons would support the old practice of implanting the divided end of the duodenum into the lower end of the partially sutured gastric incision; it may have to be done, but there is a safer way. Similarly, many surgeons do not approve of "lateral implantation" after enterectomy or colectomy, but prefer suture of the divided ends and a subsequent lateral anastomosis. Gastrostomy is a little shabbily treated perhaps, for it is questionable whether one method is always equally applicable. The excellent account of partial removal of the stomach would have gained by allusion to the method of almost complete gastrectomy practised by Moynihan. We think it very doubtful if many of the younger surgeons have a "hernia knife" among their instruments, and certainly a great many have never seen it used; surely the general practice is to divide the constricting band from without inwards. It will be seen that the criticisms we offer are almost captious; that, in itself, may be taken as a measure of the satisfaction with which we have read the majority of what is written. The book can be strongly recommended to all young surgeons; it represents an individual experience and preference, but it is sound surgery. The man who has read these pages and thought over them in the light of daily practice will find his judgment and decision surer, and will be better able to foresee and avoid accidental complications. The book is well printed and admirably produced.

Gunshot Wounds. By Major C. G. Spencer, M.B., F.R.C.S., R.A.M.C. (Oxford Medical Publications: Henry Frowde and Hodder and Stoughton. Price 5s. net.)

With the experience accumulated in the Hispano-American, Anglo-Boer, and Russo-Japanese wars on the effects of modern small-bore high-velocity firearms, a succinct text-book of moderate dimensions has for some-time been a want. The detailed reports of the medical services in these wars are too long and expensive, the narratives of individual surgeons too personal and not sufficiently confined to military surgery to supply the deficiency. Major Spencer is therefore well advised to publish in extended form the lectures which for the past three years he has given as Professor of Military Surgery at the Royal Army Medical College in London. His work embodies the conclusions and observations of those best qualified to judge of the surgical lessons taught in the three wars we have mentioned. The teaching is rational, consistent, and intelligible, and especial stress is rightly laid upon the points in which the environment of the surgeon on the battlefield modifies the principles of surgery as practised in a hospital ward. Speaking generally, the author is sparing of illustrative cases, nor is this a disadvantage: whenever he does recite one it is always both pertinent and interesting. Professionally some of the most novel lesions dealt with —novel, that is, to a civilian surgeon—are the curious concussions of nerves which sometimes result from the passage of a high-velocity bullet through adjacent tissues. The symptoms resemble, broadly, those of concussion of the brain: there is temporary loss of function without visible lesion, with subsequent complete recovery. We do not remember to have seen these peculiar cases mentioned in the current surgical text-books; but it seems only reasonable that some account of them should there appear, considering the number of medical men who enter the medical services of the Crown either in the regular or auxiliary forces. The chapter on fractures is extremely good, and illustrated by skiagrams which are well chosen and reproduced. In fact it is difficult to find any section to which like praise cannot be awarded. The work can be recommended with complete confidence to all who anticipate that they may ever be called upon to treat gunshot wounds, and that—if contemporary playwrights and many other reflecting people are right—means every qualified medical practitioner in Great Britain.

### GYNÆCOLOGY.

Text-book of Gynæcological Diagnosis. By Dr. G. Winter, Königsberg, and Dr. Karl Ruge, Berlin. A Translation after the third revised German edition. Edited by John G. Clark, M.D. (Philadelphia and London : J. B. Lippincott Co. Pp. 670. Price 25s.)

The book is, roughly, divided into three sections under the headings "Methods of Examination," "Special Diagnosis," and "Analytical Diagnosis." The second section takes up the great bulk of the work, and naturally deals with the diagnostic points of the various pathological conditions of the female genital organs under their separate headings. Analytical diagnosis, on the other hand, deals with prominent symptoms, such as hæmorrhage, amenorrhœa, dysmenorrhœa, and describes the lines along which the differential diagnosis of the causes of these conditions is to be made. The first section describes carefully the modern methods of investigation, and we are pleased to see that the use of the uterine sound is regarded as by no means a simple operation, not a "necessary part of every gynæcologic examination," and "should be used as rarely as possible." The paragraphs on the use of the cystoscope are very good and clear. The methods of investigation by removal of tissue for microscopic examination are well set forth, and throughout the book the enormous value of the microscope in gynæcological diagnosis is very properly insisted upon. In a footnote the editor mentions the Goodell-Ellinger dilator for investigating the interior of the uterus, which is much used in America. We think he would have been well advised to point out its manifest dangers and disadvantages as compared with metal forms of Hegar's dilators.

The chapters on the normal microscopic structure of the genital organs are excellent, and form a most useful basis for the histological pathology of the subject. Whilst we fully endorse the very complete description of the various forms of endometritis, we cannot think that the terrible titles coined for them by the author can be quite necessary. For instance, "endometritis glandularis ectatica cystica interstitialis chronica" is a triumph of redundancy which does not appeal to us, and, we should expect, would horrify our transatlantic brethren. We should have expected to find some more description of kraurosis vulvæ than is contained in the eleven lines devoted to it, considering it was first described in Vienna. The analytical chapters are well written, and if we cannot agree with every word in them, especially that on dysmenorrhœa, still we cannot but admire their general arrangement and the admirable method which the reader will acquire by reading them. The illustrations are for the most part very good.

Essays on the Position of Abdominal Hysterectomy in London. By J. Bland-Sutton, F.R.C.S. (London : James Nisbet and Co. Pp. 90. Price 2s. 6d. net.)

Six essays on different aspects of the operation of abdominal hysterectomy are contained within the covers of this monograph, and they are all well worthy of perusal by everyone interested in pelvic surgery. Beginning with the operation for fibroids, the author wastes no time over details of operative technique, save to emphasise the importance of the use of gloves and of celerity in operating. In the latter respect some gynæcologists think Mr. Bland-Sutton has in practice carried his principles, if anything, too far; but it is candidly to be admitted that there are those among his critics whose methods are unquestionably far too dilatory. He pronounces, on the whole, for sub-total rather than total hysterectomy, though he calls attention to the fact that he has performed even the latter operation 200 times, and is therefore familiar with its merits and the indications for it. Another section of this chapter deals with the value of a "belated ovary"—that is, one left behind by an operator performing hysterectomy; while the relation of uterine fibroids to pregnancy is also considered, especially in relation to the "red degeneration" sometimes seen when the two conditions are associated. To injuries of the ureters during the operation a whole chapter is devoted. This complication is without doubt much more common even than Mr. Bland-Sutton's statistics show. A full and satisfactory account of injuries to the uterus contains descriptions of several cases from the literature which are of very great interest and importance; and the succeeding essay upon thrombosis and embolism after operations on the female pelvic organs is also full of practical clinical wisdom. The author's position upon this point is that sepsis, though not necessarily the only cause, is certainly the chief cause of post-operative thrombosis and embolism. The very full bibliographies at the ends of the chapters increase the value of a useful and timely collection of clinical essays.

### TROPICAL MEDICINE.

Review of Recent Advances in Tropical Medicine, Hygiene, and Veterinary Science, being a Supplement to the third Report of the Wellcome Research Laboratories at the Gordon Memorial College, Khartoum. By Andrew Balfour, M.D., and R. G. Archibald, M.B., R.A.M.C. (Published for Department of Education, Sudan Government, Khartoum, by Baillière, Tindall, and Cox. 251 pp. Price 10s. 6d.)

This forms a supplement to the third Report of the Wellcome Laboratories at Khartoum. Primarily meant to assist medical officers in the out-stations of the Sudan, it will quite likely prove of equal value to others similarly situated in other colonies. Many subjects are dealt with, amongst the most important being bacteriology, climate, dysentery, fevers, hæmatozoa, leishmaniosis, liver abscess, malaria, Malta fever, mosquitoes, paratyphoid fever, piroplasmosis, plague, sleeping sickness, smallpox, spirochaetosis, sprue, syphilis, ticks, tsetse flies, tuberculosis, yaws, and yellow fever. Considerable advances have taken place of late regarding many of these complaints, and all recent work will be found in the pages devoted to these subjects. After reading through the whole review one tries to picture the state of mind of the ordinary colonial practitioner after he has perused it—it probably would be one of chaos. Tropical medicine has changed with a rapidity almost incredible, and to keep pace with it the student must be more or less a specialist on many scientific subjects, many of which will not directly affect him in his ordinary medical work in the future. Again almost three-quarters of this recent work deals with diseases of animals, which, useful to veterinary surgeons and scientists, possesses only a minor interest to students who are more concerned with human medicine and clinical studies. Though this is so it is no fault of Dr. Balfour, and one must not condemn the review because it contains so much scientific work. For the student with scientific inclinations it will form an admirable summary of the work of the last few years, and as Dr. Balfour, we think, hints, it will be excellent for students preparing for their Diploma in tropical medicine and hygiene.

### PAEDIATRICS.

Infant Feeding. By J. S. Fowler, M.D., F.R.C.P.Ed. (Oxford Medical Publications: Henry Frowde, and Hodder and Stoughton, 1909. Pp. 230. Price 5s. net.)

This work is described as a practical guide to the artificial feeding of infants. Dr. Fowler states in his Preface that he has not attempted to treat the subject exhaustively, and it is certainly a very grave defect to omit a full description of maternal nursing, the management thereof, and the indications or contra-indications for weaning. Such omissions tend to discredit the natural mode of feeding, though there may be a certain amount of justification for it, since the work is obviously written for the medical practitioner and not for the general public. In the first chapter the vital properties of milk are insisted on, and a certain space is allotted to the varieties of casein, on which the observations of Van Slyke and Hart are apparently adopted in an entirely uncritical spirit. On the composition of cow's milk Dr. Fowler appears to hold uncertain views, for he quotes Van Slyke's analysis in which the percentage of sugar is much higher than that of most chemists, refers to Raudnitz' analysis in the Appendix, and in the body of the book recommends that as a working average the percentage of sugar should be taken as four. On the heating of milk the author's views are quite sound and orthodox. Perhaps the recommendation that "boiling the milk for five to ten minutes after it is received is perfectly safe and satisfactory" is dangerous advice, for there is no warning that it should be then cooled down rapidly and kept covered up in a cool place. Too great emphasis is laid on securing a sterile food and the application of the surgical principle of asepsis to the baby's diet. For this reason he advocates cooked milk. A caution should be added to the recommendation of enamelled iron ware, for it is liable to splintering, and the chips are not satisfactory additions to the food. The diet advised during the second year is less liberal than usual in London. Dr. Fowler does not add gravy or mashed potatoes until "from the eighteenth month," nor fish, bread soaked in bacon fat, milk puddings, etc., until the latter half of the second year. Four pages on weaning and mixed feeding follows the four pages on the diet for the second year; not an orderly arrangement. These, perhaps, are trivial criticisms. The printing is clear, the paper admirable, and the bulk of the illustrations useless or unnecessary. Pictures of milk, curdled by various methods, resemble white clouds in a dusky sky. Weight charts occupy an undue space. Yet we may sum up the book as quite a valuable guide to the qualified practitioner, provided that he is prepared to fill in the gaps.

### EUGENICS AND HEREDITY.

Heredity and Disease. A Discussion opened by Sir William S. Church, Bt., K.C.B., M.D.; Sir William R. Gowers, M.D., F.R.S.; Arthur Latham, M.D.; and E. F. Bashford, M.D. (Longmans, Green and Co., 142 pages with index and diagrams.)

The exceptional difficulties which are to be met with in any attempt to consider the phenomena of heredity, are very much emphasised by the publication in book form of the discussion which took place under the ægis of the Royal Society of Medicine. Curiously enough, it is probable that the failure to arrive at any definite conclusion will be of immense value to the study of heredity. Many notable men took part in this debate, and the only inference which we can rationally draw, unfortunate though it may seem, is that the lack of accuracy and tendency to jump to irrelevant and unjustifiable conclusions is exceptionally marked in these pages. Dr. Mercier's paper, which begins with a just indictment of these fallacious methods, ends unfortunately with an acceptance of the "Mutations Theorie" of De Vries which, so far as biology is concerned, is at present a matter open to discussion. Mr. Mudge is principally concerned with an attack upon Professor Pearson, which naturally is not conducive to the furtherance of the question for debate, while Professor Pearson, who contributes two papers, is obliged to devote the second to his own defence. But, if we consider the careful statements of Sir William Gowers, of Dr. Bullock, and the other contributions of a more medical nature, we cannot fail to be impressed by one benefit which must accrue from this meeting—a more scientific attitude of medical men to the possibilities of heredity. There can be no doubt about the necessity for collecting material, both in the case of normal and abnormal members in a family; it is equally imperative to insist upon caution and reticence in the expression of inferences from the material collected. In fact, it might be suggested that the Royal Society of Medicine should consider the formation of some office to which records and genealogies might be sent, and the material and evidence thoroughly tested. At present, there does not seem to be any absolutely incontrovertible test of heredity, and until such be discovered, all statements must be made with certain reservations. Although, according to Professor Pearson, the bulk of biologists hold as untenable the inheritance of acquired characters, there is no need for the blind acceptance of their view, which is simply based upon the theories of Professor Weismann. "No disease," as Sir William Church observed, "which arises from or is associated with the presence of a foreign body, whether living or dead, within us can be considered hereditary," and the field is still open for further investigation and the evolution of some practical and applicable theory.

The Eugenics Review. (Published quarterly by the Eugenics Education Society. Price 1s. net.)

However much we may be in sympathy with the objects of this society, we must protest against one or two features which are noticeable in this review. Dr. Saleeby offends against the principles of true science by a kind of pretentiousness which is painfully obvious on page 41. He speaks of "some observations not yet published by the Mendelians, which show that there is in the hen a definite brooding instinct." Whatever Mendelism may or may not prove, it is still only an hypothesis upon which much more work remains to be done. If these observations have not been published by their author or authors, Dr. Saleeby has no right to use them in this cut-and-dried popular way. No cause is furthered by such slipshod work, whether the permission of the authors to make this statement has been obtained or not. It is clearly an attempt to acquire authority by means of a special revelation, and as such is unscientific. We cannot, on the whole, recommend this review, or, to be more accurate, we think that the review is at present superfluous. The publications of the Eugenics Laboratory are quite sufficient to bring the eugenics work before educated men, and this attempt at a popular appeal does not seem felicitous. Such a publication as the present is likely to do more harm than good, particularly if subsequent numbers contain statements of the same calibre as those made by Dr. Saleeby.

### MISCELLANEOUS ITEMS.

Medical Reports of the Central London Throat and Ear Hospital. Vol. I. (London: Adlard and Son. 1908. Pp. vi. + 134. Two plates and eight illustrations. Price 5s. net.)

The Staff of the Central London Throat and Ear Hospital having decided to issue Reports, the first volume has now duly appeared. It is not clear whether it is intended to continue these Reports regularly, but they will presumably be published at comparatively long intervals, as the cases described in the present volume go back as far as 1901. The volume contains several articles of interest to the specialist, notably one by Dr. Dundas Grant on the treatment of phlebitis of the lateral sinus, in which he points out that ligature of the internal jugular vein does not cut off all the avenues of systemic infection and should be avoided when sufficient disease is found in the sinus to explain the symptoms. The opinion which he expresses in another article, that it is advisable to leave the matrix of a cholesteatoma when it is smooth and complete, will meet with less general acceptance. There is an interesting communication by Dr. Wyatt Wingrave on the presence of acid-fast bacilli and spirochætæ in aural discharges; and some points of interest may also be gleaned from Dr. Kingsford's summary of the anæsthetics administered at the hospital. We are surprised to find from this review that all operations for tonsils and adenoids are performed in the sitting posture; ethyl chloride is now almost exclusively given, and specific mention is made of several cases under six months old; although there have been no fatalities, and although there may be little danger in very expert hands, we cannot agree that it is the safest position in which to perform this operation on young children. The volume is well worth perusal by those interested in the special subjects concerned.

International Clinics. Seventeenth Series. Vols. III. and IV. (Philadelphia and London: J. B. Lippincott Company.)

We have on several occasions had the pleasure of welcoming the successive volumes of the "International Clinics" and of commending them to the attention of our readers. The standard of excellence which we have recognised in previous issues is well maintained in the latest numbers of the series, and the contributions, as usual, embrace practically all the departments of professional activity. Even to mention the several articles is impossible in the space at our disposal, but as an indication of the wealth and variety of the fare provided an allusion to one or two contributions may be permitted. Thus in the Medical Section is a careful study of Blood-Pressure in Tuberculosis, by Dr. W. B. Stanton, and an elaborate article on Cardiac Arrhythmias and their Clinical Significance, by Dr. A. W. Hewlett; the latter paper has a special interest as the author does not adopt in all respects the interpretation of the venous pulse tracings now generally current. Other contributions which will appeal to the physician are The Action of Metallic Ferments in the Treatment of Pneumonia, by Professor Robin, of Paris, and the Treatment of Diabetes, by Dr. David L. Edsall. The Surgery of the Blood Vessels (Dr. J. E. Sweet), Contraction of the Tendo Achillis (Mr. A. H. Tubby), and Torsion of the Testicle (Mr. Edred M. Corner), are interesting features of the Surgical Section. These on the present occasion must suffice us, but there is hardly a department of professional work in which the practitioner will fail to find in these volumes something of interest and value. The Editor is to be congratulated on the attractive and serviceable papers he is able to submit to the profession.

A History of the Reading Pathological Society. By Jamieson B. Hurry, M.A., M.D. (London: John Bale, Sons and Danielsson, Ltd. With 10 Illustrations. Pp. 179. Price 7s. 6d. net.)

In the volume before us the President of the Reading Pathological Society gives a short account of the history and vicissitudes of one of the oldest and most vigorous of provincial medical societies, from its inception in 1841 to the present day. Founded by a number of practitioners in the locality, the society owed much to the encouragement and practical assistance of the Governors of the Royal Berkshire Hospital, who from the commencement placed a room in the hospital at the disposal of the members for their meetings. Throughout its career of nearly seventy years the society seems to have maintained a high level of distinction, both as to the quality of the papers read at its meetings, and as to the attainments of its members and the orators who have been honoured with the task of reading the annual address. These latter comprise many whose names are pre-eminent in the medical world. The author's task has evidently been a labour of love. He sets forth a summary of each session with a list of the papers read, cases shown, and pathological specimens exhibited. A number of portraits of officers of the society are scattered through the book, which is completed by a series of appendices, which include a catalogue of the specimens in the society's pathological museum and a description of its library. The author's enthusiasm, where it occasionally runs to excess, will no doubt be forgiven by his fellow-members, who owe him a debt of gratitude for his exhaustive labours on behalf of their society.

The Sportsman's Guide to North-Eastern Rhodesia. By J. Dunbar-Brunton. (London: The Scientific Press, Ltd. 1909. Price 2s. 6d. net.)

This brief monograph contains a good deal of excellent advice in small compass. Its scope is sufficiently indicated by the title, but as a matter of fact the greater part of the contents will apply to any part of tropical Africa which is visited by big-game shooters. As it is intended for laymen the hints on the preservation of health are not of the comprehensive nature which would be expected of a medical manual; but they are sound as far as they go. The very brief vocabulary of useful words in three native languages might with advantage have been somewhat longer. An excellent map is provided, and probably accounts for the price at which the book is published. Any medical man whose lot takes him to this distant region can be advised to pack this guide among his kit. If anything, we think the author might have been rather more liberal also in notes on the big game of this province.

The "Nauheim" Treatment of Diseases of the Heart and Circulation. By Leslie Thorne Thorne, M.D. (London: Baillière, Tindall, and Cox, 1909. Third Edition. Price 3s. 6d. net.)

The fact that this book has reached a third edition testifies to its popularity. It fully merits the reception it has had. The main object of the work is to indicate succinctly how any medical practitioner in England can carry out the details that are essential to the Schott-Nauheim treatment by baths and by graduated exercises without sending his patients away at all. The excellent illustrations of the various resisted movements are a great feature of the book; they make each exercise as easy to follow as can be. We strongly recommend the work to every practitioner.

